# Paternal and Maternal Influence on Delinquency among Early Adolescents in Hong Kong

**DOI:** 10.3390/ijerph16081338

**Published:** 2019-04-14

**Authors:** Daniel T. L. Shek, Xiaoqin Zhu

**Affiliations:** Department of Applied Social Sciences, The Hong Kong Polytechnic University, Hong Kong, China; daniel.shek@polyu.edu.hk

**Keywords:** junior high school, Chinese students, delinquency, longitudinal, family

## Abstract

*Objective*: The aim was to examine the effects of parental behaviors and the parent-child relationship on delinquency levels as well as growth rates among early adolescents, and to explore the cross-sectional and longitudinal influence of fathers and mothers. *Method*: The study used and analyzed data collected at Waves 1–3 (*N* = 2669, age 12.56 ± 0.71 years at Wave 1) in a six-year research project. *Results*: While both parents’ behavioral control significantly predicted a lower initial level of delinquency, only higher behavioral control of fathers predicted a fast increase in delinquency. In contrast, parental psychological control did not serve as significant predictors in the individual growth curve model. Besides, relationships of father-child and mother-child dyads negatively predicted the initial level of delinquency but not the rate of change in adolescent delinquency. When all factors were investigated simultaneously, fathers’ behavioral control and the relationship between mother and child were robust cross-sectional predictors, whereas only the latter was a stable longitudinal predictor of adolescent delinquency. *Conclusions*: Parenting and the parent-child relationship are predictors of adolescent delinquency. It is necessary to differentiate between: (1) adolescent delinquency level and its change rate over time; (2) different aspects of parent-child dyadic factors; and (3) paternal and maternal factors.

## 1. Introduction

Empirical findings suggest that children’s psychosocial and behavioral development is largely shaped by familial factors. While favorable family environment and favorable parenting characteristics such as responsiveness, warmth, and consistent discipline serve as protective factors, negative parental behaviors such as rejection, hostility, and psychological control act as risk factors [1,2]. Nevertheless, there are shortcomings in the extant literature. First, limited research has considered parental impacts from multiple aspects simultaneously. For instance, very few studies have investigated parents’ behavioral and psychological control and the parent-child relationship together. Second, the vast majority of previous studies considered parenting characteristics from one parent or did not distinguish between paternal and maternal impacts. Third, compared with numerous studies investigating parental impacts on the level of delinquency, empirical efforts linking parental factors with the growth rate of adolescent delinquency in a longitudinal study are grossly inadequate [3]. Therefore, this study attempted to address these research gaps among Hong Kong Chinese adolescents by using a 3-year longitudinal research design. 

### 1.1. Adolescent Delinquency and Its Developmental Trajectory

Adolescence is a challenging transition period characterized by physical, psychological, and social changes as well as various pressures such as academic and peer pressures. If adolescents are not able to adequately cope with these developmental challenges, problematic behavior such as delinquency can occur. Abundant evidence has revealed a relatively high incidence of delinquency during adolescence worldwide. For example, a study using a representative sample showed that 46% of adolescents in Grade 7 to 12 in the United States engaged in offending behavior [4]. A lower but still a significant incidence of adolescent delinquency has also been observed in Asia, for example in Hong Kong and South Korea [5,6]. 

Delinquent behavior level among youth appears to vary with their age, typically taking an inverted U-shaped trajectory. Specifically, the level of delinquent behavior increases when individuals develop from childhood to early adolescence and reaches its peak gradually (i.e., the rate of increase slows down as adolescents age), then declining from late adolescence to adulthood. Such a developmental route has been observed in different cultures [1,3,7,8]. For instance, it was identified that the delinquency level was lower in early adolescents than that in mid-adolescents, while late adolescents demonstrated more delinquent behaviors than did adults [9]. In addition, Shek and Yu [7] tracked Hong Kong Chinese adolescents’ delinquency from Grade 7 to 11 through eight occasions of assessment. The authors found an increasing trend with the rate of growth declining over time. Recently, Cho [1] observed the similar change pattern of delinquent behavior among South Korean adolescents. 

Delinquency brings disruptive influences on adolescent future development, family dynamics, school performance, and even the stability of society. Essentially, early and persistent delinquent acts have been identified as a robust predictor of later violent behavior, partner conflict, unemployment, and health risk behavior including substance abuse and dependence [10,11]. A more recent study utilizing longitudinal data found that the more frequently adolescents engaged in petty theft and fighting, the more likely they were to drop out of high school and report depression symptoms and substance abuse problems in early adulthood [12]. It is undoubted that all families and communities have to bear these significant costs, and serious consequences result from adolescent delinquent behavior [13].

Given its high incidence, especially during early adolescence, and its long-term adverse consequences, adolescent delinquency has become a critical social problem. In order to further the understanding of adolescent delinquency and facilitate the development of evidence-based prevention programs and/or interventions, scholars have devoted many efforts in delineating factors that significantly impact adolescent delinquent behavior. Although no single factor has been claimed to be able to explain the most variance of adolescent delinquent behavior, decades of research suggest that parental factors are among the most influential ones [2,14].

### 1.2. Influence of Parental Control 

In literature, family processes have been commonly emphasized as utmost important factors that influence adolescent delinquent behavior, because family constitutes the basic ecology where children are socialized and their behavioral patterns are shaped [15]. For example, positive family functioning, parental supervision, and attachment exhibited negative associations with adolescent delinquent behavior [6,16,17], while neglect and a poor parental–child relationship positively predicted adolescent delinquent behavior [8,18]. Among different parental factors, parental control has been emphasized as one critical dimension that is vital to the development of adolescent delinquency [2].

Parental control is a multidimensional concept that includes two constructs: behavioral and psychological control [19]. Behavioral control represents parents’ endeavors to regulate and manage their children’s behavior by means of guiding them to behave in a socially acceptable way, setting rules and restrictions, and monitoring their activities. Because behavioral control indicates consistent discipline and active surveillance practiced in a warm manner, it is instrumental in protecting children against problem behaviors and leads to favorable developmental outcomes [20]. In particular, scholars regarded delinquency as an extensive representation of a child’s natural impulsivity and hedonism and contended that children need to learn not to commit delinquency rather than learning to commit it [21]. As a result, direct behavioral control makes parents knowledgeable about children’s whereabouts and conduct, enabling them to communicate with children about what behavior is unacceptable and curb future delinquency in children [17]. Empirical findings support these arguments by consistently showing that parental behavioral control and its related components such as monitoring and parental knowledge are negatively associated with children’s delinquency and other problems [2,22,23]. For example, a recent meta-analysis showed that parental behavioral control had negative cross-sectional and longitudinal associations with delinquency and other forms of externalizing problems [24].

In contrast, *psychological control* attempts to manipulate children’s behavior and control their thoughts, feelings, and emotions through insidious behaviors such as guilt-inducing, love withdrawal, personal attack, and disrespectful parenting. Psychological control brings intrusions into children’s development and harms their well-being. As remarked by Rogers et al. [25], “psychological control communicates that the adolescent’s thoughts, emotions, feelings, and/or even the adolescent are unacceptable” (p. 350). Indeed, such intrusive parenting has been rated as a violation of children’s self-representation concepts (e.g., self-efficacy, self-identity, and self-esteem) and sense of autonomy [26], which subsequently lead to children’s dysfunctional coping strategies such as externalizing behaviors or internalizing problems. Furthermore, such intrusive parenting may be a manifestation of the parents’ own psychopathology, such as depressive symptoms and anxiety [27,28], considered as important factors for increased risk of emotional and behavioral maladaptation in terms of delinquency and other problems in children [29,30]. Consistent with these studies, there are rich empirical findings showing that psychological control of parents significantly elevates the risk of having externalizing problems including delinquency among children [2,24,31,32]. 

Despite the conclusive findings across cultures that parental behavioral control protects children against delinquent behavior while psychological control positively contributes to adolescent delinquency [33], several shortcomings of the existing literature deserve attention. 

### 1.3. Research Gaps 

The first issue is that inadequate research has investigated parental control together with parent-child relationship quality which constitutes another important aspect of the family process [34]. A good parent-child relationship is usually characterized by intimate emotional bonding, good communication, trust and satisfaction between a child and his or her parents [34]. A negative linkage between parent-child relationship quality and adolescent delinquency can be formed through two ways. First, it is believed that a good parent-child relationship makes a child strongly attached to the parents, interpret parental discipline in a positive manner, and comply with social norms and values advocated by parents and behave accordingly [35]. This socialization process can be regarded as involving “indirect parental control” that mitigates adolescent delinquent behavior [17,36]. In addition, considering Donald Woods Winnicott’s perspective on antisocial tendency and acts, it is possible that children’s delinquency communicates their hope and needs to get responses from parents and to search for nurturing relationships with parents, which have instinctively felt by the children to be missing [37,38]. This theory also implies a negative relationship between the parent-child relationship and delinquency.

Second, a high-quality parent-child relationship creates a trustful and secure environment for children to openly communicate with parents and voluntarily disclose themselves, facilitating parental behavioral control and subsequently reducing adolescent delinquency. During adolescence, parental control cannot simply be parents’ active control over children (for example through tracking and surveillance) because adolescents require more behavioral autonomy and devote more time in other social relations (e.g., peers) rather than with parents [39]. Instead, parents’ awareness of the whereabouts and conduct of their children relies upon children’s self-disclosure of the information, which is more likely to happen if a good parent-child relationship exists. Indeed, open parent-child communication and more adolescent self-disclosure were associated with less adolescent delinquent behavior [2,40]. In addition, without a good parent-child relationship indicated by trust and communication, parental behavioral control could even harm adolescent development and increase delinquent behavior [41]. Given the unique role played by parent-child relationship in reducing adolescent delinquent behavior, it is important to involve it when investigating the influence of parental control. 

The second research gap is related to the differential maternal and paternal impacts on adolescent delinquency. Hoeve and collaborators [2] remarked that “studies generally focus on one parent or both parents without differentiating between the sex of the parent” (p. 765). First of all, most research has concentrated on maternal impact and overlooked fathers’ influence, although paternal factors have demonstrated a significant predictive effects on children’s delinquent behavior and other developmental outcomes [42,43]. It was estimated that less than one-fifth of the extant literature about how parental characteristics affect youth delinquency concentrated on fathers [44]. Although fathers usually devote less time looking after their children, quality rather than quantity of father-child interaction has been argued to act as a stronger precursor of children’s developmental outcomes [45]. Given that parenting by fathers is inadequately researched, it is important to consider paternal impact in addition to maternal impact.

On the other hand, many studies did not separate maternal and paternal parenting. Instead, most studies just considered overall parenting indexed by children’s perceptions of control, trust or support from their parents [1,41,46]. Such an undifferentiated investigation of parental impacts is unable to delineate the complex dynamics and differences between father-child and mother-child dyads [47]. As a result, scholars call for more studies to distinguish between fathering and mothering and compare each other’s influence on adolescent development [2]. Recently, researchers have started to compare maternal impact with paternal impact. However, the findings are equivocal. 

Some studies found the similar influence of fathers and mothers on children’s development [48,49], while other studies found that impacts of mothers were stronger than fathers’ on their children [50,51] or that parental parenting was a stronger predictor than maternal parenting [32,44]. For example, whereas Davidov and Grusec [48] reported a similar role of maternal and paternal support in shaping children’s externalizing behavior, other studies found that mothers’, but not fathers’, behavioral and psychological control significantly predicted children’s problem behavior [50,52]. In contrast, Hoeve et al. [2] concluded that fathers’ support was more significantly linked to adolescent delinquent behavior than mothers’ support. Likewise, psychological control and knowledge of fathers rather than that of mothers were found to significantly account for variance in adolescent externalizing problems [32]. In short, despite the growing recognition of differential effects of fathers’ and mothers’ parenting, the extant findings are far from conclusive. 

The final issue has to do with the dearth of research which considers parental impacts in relation to developmental trajectory of adolescent delinquency. The vast majority of existing research was cross-sectional or only had two occasions of assessment [2], which makes it difficult to conclude the long-term effects of parenting as well as the potential influence of parenting on the growth rate of adolescent behavior. In contrast, multi-wave research enables modeling of individual growth curve for adolescent behavior, estimation of intraindividual change (i.e., the growth rate), and investigation of parental factors’ predictive effect of the interindividual differences in the growth rate [3]. Several pieces of evidence have informed possible longitudinal effects of parenting and its association with the rate of change in adolescent delinquency. For instance, Kerr et al. [53] found that active parental monitoring was not a significant predictor of changes in adolescent delinquent behavior over two years, but adolescent self-disclosure was. Besides, parental behavioral control tended to halt the growing trend of adolescent delinquent behavior [3]. Likewise, Yoder et al. [8] showed that a better father-child relationship was associated with a deeper decline trajectory of adolescent delinquency. 

The above studies have partially addressed the third knowledge gap. In addition, related findings are in line with the general theoretical prediction that favorable parenting characteristics will predict a faster decrease or a slower increase in adolescent problem behavior, which represents a positive adjustment in a long run [23]. However, these studies did not compare paternal and maternal impacts with multiple parental characteristics being taken into account simultaneously. Thus, it still remains inconclusive as to whether and how maternal and paternal factors in different aspects (e.g., behavioral and psychological control as well as relationship quality) predict the change rate of adolescent delinquent behavior. 

### 1.4. The Present Study

To address the existing research gaps, this study explored whether three parenting characteristics (i.e., behavioral and psychological control as well as the parent-child relationship) predict the levels of adolescent delinquency, both concurrently and longitudinally. Moreover, how these parental characteristics predict the growth rates of adolescent delinquency across junior high school years was also examined. 

Specifically, our study answered three research questions. The first question was as follows: “Do parental behavioral and psychological control and parent-child relationship qualities predict children’s initial levels of delinquency when they enter junior high school?” Previous findings suggest a negative linkage between positive parental factors (e.g., behavioral control, good relationship) and the incidence of adolescent problem behaviors, while negative parental factors (e.g., psychological control) lead to a higher prevalence of adolescent problem behaviors [2,23,32]. Thus, the first hypotheses were as follows: Paternal and maternal behavioral control negatively predict the initial level of adolescent delinquency (Hypotheses 1a and 1b). In contrast, parental psychological control would have positive predictive effects (Hypothesis 1c for fathers’ psychological control and Hypothesis 1d for mothers’ psychological control). Similar to behavioral control, parent-child relationship quality was also expected to negatively predict adolescent baseline delinquency (Hypotheses 1e and 1f for the father– and mother-child relationship, respectively). 

The second question we asked was: “How do the various factors of parent-child subsystem qualities predict the growth rate of adolescent delinquency across junior high school years?” Based on the general theoretical stand and limited evidence showing that favorable parental factors lead to a faster decline or a slower increase in adolescent problem behaviors [3,8], we hypothesized that stronger behavioral control of fathers (Hypothesis 2a) and mothers (Hypothesis 2b) would predict a slower increase in children’s delinquency. In contrast, fathers’ (Hypothesis 2c) and mothers’ (Hypothesis 2d) stronger psychological control would predict a faster growth rate of children’s delinquency. Finally, better relationships between children and their fathers (Hypothesis 2e) and mothers (Hypothesis 2f) would predict a slower increasing rate in adolescent delinquency. 

The third question we addressed was: “What are the concurrent and longitudinal contributions of different parental factors to adolescent delinquent behavior?” Existing theories and empirical findings were inconclusive regarding the relative paternal and maternal impacts on adolescent development [32,44,50,51]. Besides, limited research has considered multiple parental factors while differentiating paternal and maternal factors using longitudinal data [23]. Against this background, we set two competing hypotheses: Paternal factors are more influential than maternal factors in shaping adolescent delinquency (Hypothesis 3a); and maternal factors are more influential than paternal factors in influencing adolescent delinquency (Hypothesis 3b). Table 1 shows the above-mentioned hypotheses. 

We addressed the questions by using a large-scale sample of Chinese adolescents over the three-year junior high school stage in Hong Kong. In contemporary Chinese families, parenting and children’s socialization processes are jointly shaped by traditional Chinese philosophies (e.g., collectivism) and Western values (e.g., individualism and gender equality) [54]. This is especially true in Hong Kong where a relatively Westernized and industrialized subculture has developed due to the special history of Hong Kong. The adherence to traditional Chinese culture may result in unique findings regarding the research questions of the present study. For example, the traditional “strict father, kind mother” model assumes paternal responsibility of disciplining (“guan”, 管) children and maternal warmth and emotional closeness [54], which may suggest a stronger effect of fathers’ behavioral control and greater influence of mother-child relationship. However, such a conclusion is not necessarily the case because the traditional belief has been shifting to “strict mother, kind father” with the ongoing social transitions and development [54,55]. Thus, it will be interesting to examine related questions within this unique social and cultural context.

In this study, children’s gender effect is also considered. Overall speaking, boys show a higher level of delinquency as compared to girls as boys are hypothesized to be not only more vulnerable to risk factors but also exposed to risk factors more than girls [2,24]. In this sense, parenting effect may be weaker among girls due to the higher delinquency in boys. However, related studies were insufficient and findings were inconsistent [2,24]. Specifically, while some studies reported stronger parenting effects on boys, some others found a very minimal moderating effect of children’s gender on the association between parenting and children’s delinquency level. By considering children’s gender in addition to parents’ gender, the present study contributed to the ongoing investigation as to whether fathers and mothers exert equivalent influence on sons and daughters.

## 2. Materials and Methods

### 2.1. Participants and Procedures

We used data collected in a 6-year longitudinal research project launched in the 2009–2010 school year, which involved more than 3000 students in 28 Hong Kong secondary schools. More information on the project is outlined elsewhere [23,56,57]. The project was reviewed and approved by the Human Subjects Ethics Sub-Committee at a public university in Hong Kong (Hong Kong Polytechnic University). All related parties including adolescent participants, schools, and parents gave well-informed written consent prior to the commencement of the research project. 

Among the 3328 students (age 12.59 ± 0.74 at Wave 1) who completed the survey at the first wave (Wave 1), 2905 students completed the survey at Wave 2 (attribution rate = 12.7%) and 2860 completed the questionnaires at Wave 3 (attrition rate = 14.1%). Across first three waves (Waves 1–3) during the junior secondary school stage (i.e., Grades 7–9), 2669 student participants were successfully matched and this matched sample was utilized in this study. The time interval between two consecutive waves was one year. The mean age of the matched sample was 12.56 ± 0.71 years at Wave 1. Among the participants in the matched sample, 1321 (49.5%) were boys, 1344 (50.4%) were girls, and 4 students (0.01%) did not indicate their gender information.

The matched sample (*N* = 2669) and the 659 students who withdrew from the study after Wave 1 (i.e., dropouts) were compared regarding their basic demographic profile, baseline delinquency level and parent-child subsystem qualities. It was found that the matched sample consisted of a higher percentage of girls (Chi square (degree of freedom is 1) = 39.70, *p* < 0.001, effect size (*φ*) = 0.11). Besides, the mean age of the matched sample (age 12.56 ± 0.71 at Wave 1) was slightly younger than that of the dropouts (age 12.72 ± 0.86 at Wave 1, *F*_(1, 3233)_ = 35.52, *p* < 0.001, *η*^2^ = 0.02). With reference to delinquency, the matched sample showed a lower baseline level (*F*_(1, 3115)_ = 50.54, *p* < 0.001, *η*^2^ = 0.01). While no significant difference was found in father’s behavioral control (*F*_(1, 3190)_ = 0.26, *p* = 0.61), a slightly lower baseline level of both parents’ psychological control, a slightly higher baseline level of mother’s behavioral control and better relationships with both parents were observed among the matched sample (*F* values ranged from 5.40 to 35.52, *p*-values were <0.05, and *η*^2^ ranged from 0.002 to 0.01). As the effect size was not large in the observed differences, it was concluded that attrition is not a major problem in this study. 

### 2.2. Instruments

The project used a questionnaire including multiple measures [58], among which adolescent delinquency and the three indicators of parent-child subsystem qualities were the focus of this study. 

Delinquency was assessed using a validated scale measuring the frequency of participants’ involvement in delinquent behavior (i.e., “How often do you have each of the twelve delinquent acts listed below during the past one year?”) [59,60]. The delinquent acts included “stealing”, “cheating others”, “truancy”, “running away from home”, “damaging others’ properties”, “beating others”, “gang fighting”, “speaking foul language”, “having sex with others”, “staying away from home overnight without parental consent”, “bullying”, and “trespassing”. Participants gave their answers on a 7-point reporting scale (0 = “never”; 6 = “more than 10 times”). In this scale, delinquent behavior was defined as actions that harm others or break certain rules, regulations or norms. Of note, these delinquent acts consisted of both illegal behavior and minor offences that are considered risk with reference to family, school, and social norms. The scale showed acceptable construct validity and reliability in previous studies [59,60]. In the present study, confirmatory factor analyses showed that the scale was unidimensional and had adequate factorial validity and reliability across the three waves as indicated by the mean factor loading (over 0.50), average variance extracted (over 0.25), and composite reliability (over 0.70) for details, see [56]. Besides, this scale showed acceptable internal consistency at Waves 1–3, with Cronbach’s α ranging from 0.79 to 0.84 and mean inter-item correlation coefficients ranging from 0.25 to 0.33 (see Table 2). Furthermore, item-total correlations of the 12 items exceeded 0.20 across waves. Given these reliability figures, adolescent delinquency was indicated by the mean score of the 12 items in the present study.

Parent–Child Subsystem Qualities were measured by a Chinese scale entitled Parent-child Subsystem Quality Scale (PCSQS). The scale has been validated among Hong Kong Chinese adolescents and demonstrated good reliability in previous studies [61,62,63]. The PCSQS included two subscales pertinent to paternal and maternal factors respectively. Three aspects of parental factors were measured by 17 items in each subscale. First, behavioral control subscale included seven items (e.g., “My father/mother asked me about what I did after school”). Second, the subscale of psychological control consisted of four items (e.g., “Dad/Mom often wants to change my mind or feelings for things”). Finally, the remaining six items belonged to parent-child relationship subscale (e.g., “I shared my feelings with my father/mother”). A 4-point measuring scale (1 = “strongly disagree”, 4 = “strongly agree”) was used. As shown in Table 2, the Cronbach’s α across different measures included in the PCSQS ranged between 0.80 and 0.91 across different time points, indicating good internal reliability of the measures.

### 2.3. Data Analysis Plan

First, we performed reliability, descriptive, and correlational analyses for measures of delinquency and parental factors. Second, to address the first and the second research questions, individual growth curve (IGC) models were analyzed to examine how parental factors predict the initial level of adolescent delinquency (i.e., the first question) and the change rate of adolescent delinquency (i.e., the second research question). Similar to IGC models tested in previous studies [23,64], time was the Level-1 predictor (i.e., within-subjects variable), which was nested into the Level-2 predictors (i.e., between-subject variables) including both control variable (i.e., gender) and parental factors. The values of time were set as “0”, “1”, and “2” at the three consecutive waves, respectively. Finally, four 2-level IGC models were analyzed and compared:

- Model 1: “the unconditional mean model” which did not include Level-1 or Level-2 predictor;
- Model 2: “the unconditional linear growth model” that only involved time as the Level-1 predictor;
- Model 3: “the linear growth model” that further included gender as a Level-2 predictor (covariate);
- Model 4: “the linear growth models” that further included all the parental factors as key Level-2 predictors. Specifically, Model 4a, 4b, and 4c tested parents’ behavioral control, psychological control, and parent-child relationship quality, respectively.

Gender–based IGC models were also tested to examine potential moderating effect of children’s gender on parenting influences. In this study, model fit was marked by three frequently used indices: the −2log likelihood, Akaike information criterion (AIC), and Bayesian information criterion (BIC). For all the indices, a smaller value informs a better model fit. To facilitate IGC modeling, gender was coded as follows: “female” = “−1”, “male” = “1”. In addition, parental factors were standardized. Furthermore, to reduce the distribution skewness of adolescent delinquency, a natural logarithmic transformation was conducted prior to IGC analyses.

Finally, we carried out several multiple regression analyses to investigate the relative contributions of paternal factors and maternal factors to adolescent delinquency (i.e., the third research question). Parental factors’ cross-sectional predictive effects were examined at each wave. For longitudinal predictive effects, we investigated how baseline parental factors measured at Wave 1 predict later adolescent delinquency behavior measured at Wave 2 and Wave 3. Furthermore, to explore moderating effect of children’s gender, the interactions between each parental factor and children’s gender were tested in the regression analyses.

## 3. Results

### 3.1. Correlations among Variables

As shown in Table 3, behavioral control of both parents as well as relationships between children and two parents were negatively correlated with adolescent delinquency. Besides, parental psychological control was generally linked with children’s delinquency in a positive manner, despite some insignificant correlation coefficients. Overall, these results are consistent with the general expectations. In addition, across the three waves, male adolescents showed a higher level of delinquency as compared with female peers. 

### 3.2. Developmental Trajectory of Adolescent Delinquency and Gender Effect

As shown in Table 4, Model 1 suggested a large intra-class correlation coefficient (ICC) of 0.535, meaning that 53.5% of the variance in adolescent delinquency was attributable to individual differences. Thus, IGC models are required to test predictors at both Level 1 and Level 2. Compared with Model 1, Model 2 including time as a predictor had a better model fit (Δ*χ*^2^_(3)_ = 160.40, *p* < 0.001, ΔAIC = 154.40, ΔBIC = 133.44). Results of the model indicated that adolescent delinquency level increased gradually over time (*β* = 0.024, *p* < 0.001).

**Figure 1:**
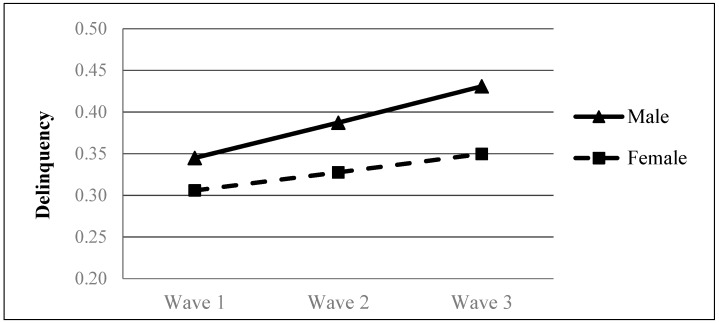
Growth trajectories of adolescent delinquency as a function of gender. The figures were plotted based on Model 3 shown in Table 5.

When gender was considered as a Level-2 covariate in addition to time (i.e., Model 3), model fit increased significantly (Δ*χ*^2^_(2)_ = 29.93, *p* < 0.001, ΔAIC = 25.93, ΔBIC = 11.96). Results depicted in Table 5 suggested that gender acted as a significant predictor of the initial level of (*β* = 0.014, *p* < 0.05) and the rate of change (*β* = 0.007, *p* < 0.05) in adolescent delinquency. Compared with female students, male students exhibited a higher level of delinquency at Wave 1 and a faster increase in delinquency over time (see Figure 1). Additionally, gender’s predictive effects remained significant after the parental factors were further included in the models (see Table 5, Table 6 and Table 7).

Further gender–based analyses for Model 2 indicated that delinquency level increased significantly among boys (*β* = 0.03, *p* < 0.001) and girls (*β* = 0.02, *p* < 0.001, see Table 4). The results suggest that the growth curve of delinquency was similar among male and female adolescents.

### 3.3. Parental Impacts on the Initial Level of Adolescent Delinquency 

The three aspects of parental characteristics were investigated in Model 4a, 4b, and 4c, respectively. Gender–based IGC models including Model 4a, 4b, and 4c yielded similar predictive effects of each parenting factor on the initial delinquency level as well the rate of change among boys and girls. Thus, in the rest of the paper we focus on the findings of IGC models based on the full sample.

As shown in Table 5, Table 6 and Table 7, comparisons of the model fit indices indicated that Model 4a (Δ*χ*^2^_(4)_ = 216.53, *p* < 0.001, ΔAIC = 208.53, ΔBIC = 180.58) and 4c (Δ*χ*^2^_(4)_ = 263.54, *p* < 0.001, ΔAIC = 255.54, ΔBIC = 227.59) had better model fit than did Model 3, suggesting that parental behavioral control and quality of parent-child relationship should be included in the IGC models. However, Model 4b failed to fit data significantly better than Model 3 (Δ*χ*^2^_(4)_ = 13.17, *p* < 0.05, ΔAIC = 5.17, ΔBIC = −22.78), indicating that parental psychological control did not add to Model 3 in explaining the variance in adolescent delinquency (i.e., parental psychological control did not serve as significant predictors in the IGC model). As such, findings did not support Hypothesis 1c, 1d, 2c, and 2d, which stated the hypothesized effects of parents’ behavioral control.

Results showed that higher levels of paternal (*β* = −0.052, *p* < 0.001) and maternal (*β* = −0.026, *p* < 0.001) behavioral control predicted a lower level of initial adolescent delinquency at Wave 1 (see Model 4a in Table 5). Thus, Hypotheses 1a and 1b were supported. Similarly, better father-child (*β* = −0.039, *p* < 0.001) and mother-child (*β* = −0.046, *p* < 0.001) relationships predicted a lower level of initial adolescent delinquency at Wave 1 (see Model 4c in Table 7). These results supported Hypothesis 1e and 1f.

### 3.4. Parental Impacts on the Rate of Change in Adolescent Delinquency

**Figure 2:**
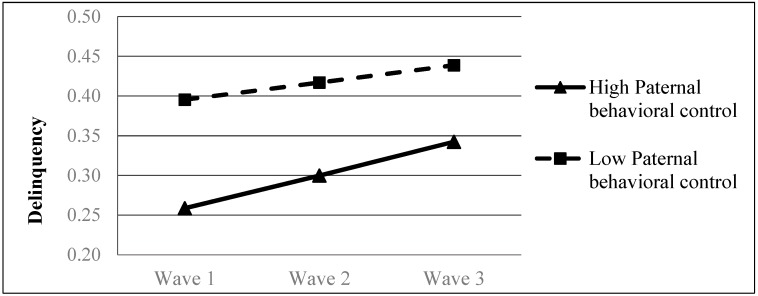
Growth trajectories of adolescent delinquency as a function of paternal behavioral control. The figures were plotted based on Model 4a shown in Table 5. High level indicates 1SD higher than the mean value; low level indicates 1SD lower than the mean value.

Results showed that paternal behavioral control was significantly associated with the rate of change in adolescent delinquency (*β* = 0.0085, *p* < 0.01) while maternal behavioral control was not (*β* = 0.0006, *p* > 0.05) (see Model 4a in Table 5). Adolescents who were under a higher level of paternal behavioral control demonstrated a faster increase in delinquency over time (see Figure 2). However, these adolescents still showed a lower level of delinquency across the three time points than their peers who were under a lower level of paternal behavioral control. The direction of predictive effect of paternal behavioral control was just the opposite to our expectation. Thus, findings did not support Hypothesis 2a and 2b. 

As shown in Table 7, quality of father-child (*β* = 0.0018, *p* > 0.05) and mother-child (*β* = 0.0054, *p* > 0.05) relationships were not significant predictors of the growth rate of adolescent delinquency. Thus, results did not support Hypothesis 2e nor 2f. 

### 3.5. Concurrent and Longitudinal Effects of Parental Factors

Results of multiple regression analyses are shown in Table 8 and Table 9, which demonstrate cross-sectional and longitudinal predictive effects of different parental factors, respectively. Further analyses did not yield significant interactions between each parenting factor and gender for both the concurrent and longitudinal effects. Thus, we elaborated results of regression analyses based on the full sample.

First, as shown in Table 8, after controlling for gender effect, the three paternal factors accounted for 7.1%, 7.9%, and 5.4% of the total variation in adolescent delinquency at three waves, respectively. Fathers’ behavioral control (*β* ranged between −0.17 and −0.16, *p* < 0.001, Cohen’s *f*^2^ ranged between 0.014 and 0.015) and father-child relationship quality (*β* ranged between −0.07 and −0.14, *p* < 0.05, Cohen’s *f*^2^ ranged between 0.003 and 0.011) showed negative cross-sectional effects at all waves, while paternal psychological showed positive effects across waves (*β* ranged between 0.05 and 0.08, *p* < 0.05, Cohen’s *f*^2^ ranged between 0.003 and 0.005). Regarding the three maternal factors, they uniquely accounted for 6.1%, 6.8%, and 4.9% of the total variation in adolescent delinquency at Waves 1–3, respectively. Similar to paternal factors, mothers’ behavioral control and their relationship with children tended to be negative cross-sectional predictors while their psychological control exerted a positive predictive effect.

Second, as depicted in Table 9, in total 4.8% (Wave 2) and 3.4% (Wave 3) of the total variation in adolescent delinquency was uniquely accounted by baseline paternal factors. For adolescent delinquency at Wave 2, all the three baseline paternal factors showed significant predictive effects (Cohen’s *f*^2^ ranged between 0.002 and 0.008). However, only father-child relationship (*β* = −0.14, *p* < 0.001, Cohen’s *f*^2^ = 0.010) was a significant predictor for children’s delinquency at Wave 3. Meanwhile, 4.6% (Wave 2) and 3.0% (Wave 3) of the variance in adolescent delinquency were uniquely explained by the three baseline maternal factors. To illustrate, baseline maternal behavioral control (*β* = −0.06, *p* < 0.05, Cohen’s *f*^2^ = 0.002) and mother-child relationship quality (*β* = −0.16, *p* < 0.001, Cohen’s *f*^2^ = 0.013) significantly predicted adolescent delinquency one year later, while only mother-child relationship (*β* = −0.14, *p* < 0.001, Cohen’s *f*^2^ = 0.010) significantly predicted adolescent delinquency in two years.

Third, all the cross-sectional parental factors uniquely explained 9.0%, 10.0%, and 6.8% of the total variation in adolescent delinquency at Waves 1–3, respectively (see Table 8). Among all factors, fathers’ behavioral control (*β* ranged between −0.15 and −0.13, *p* < 0.001, Cohen’s *f*^2^ ranged between 0.008 and 0.012.) and quality of mother-child relationship (*β* ranged between −0.17 and −0.07, *p* < 0.001, Cohen’s *f*^2^ ranged between 0.002 and 0.015.) were two significant concurrent predictors of adolescent delinquency at all waves. With reference to longitudinal predictive effects depicted in Table 9, all baseline parental factors uniquely accounted for 6.1% and 4.6% of the total variation in later adolescent delinquency measured at Waves 2 and 3, respectively. Although the three baseline paternal factors and mother-child relationship quality tended to have longitudinal effects on adolescent delinquency at Wave 2, only father– and mother-child relationship qualities showed significant predictive effects at Wave 3 (father: *β* = −0.10, *p* < 0.01, Cohen’s *f*^2^ = 0.005; mother: *β* = −0.11, *p* < 0.01, Cohen’s *f*^2^ = 0.006). 

It is noteworthy that paternal factors accounted for a relatively higher percentage of total variation in concurrent adolescent delinquency. However, for longitudinal effects, father related and mother related factors at Wave 1 explained an equal amount of variance in future adolescent delinquency. As such, our findings favor Hypothesis 3a more than Hypothesis 3b regarding cross-sectional effect. 

Further comparisons between regression coefficients at Waves 1 and 2 showed that, while paternal behavioral control had significant greater concurrent predictive effects than maternal behavioral control (absolute value of z (|*z*|) > 1.96, *p* < 0.05), mother-child relationship quality tended to have greater concurrent effects than father-child relationship quality (|*z*| > 1.96, *p* < 0.05). Such differences also presented for the longitudinal effects on adolescent delinquency at Wave 2 but did not exist for the cross-sectional or longitudinal effects at Wave 3. These results suggest that specific aspect of parent-child subsystem should be considered when investigating relative paternal and maternal impacts.

## 4. Discussion 

The current study investigated the influence of three parental characteristics on the development of adolescent delinquency among Hong Kong Chinese adolescents during the junior high school years. We addressed three research questions in this study. First, we separately investigated paternal and maternal impacts on the initial level of adolescent delinquency. Three aspects of parental characteristics were referred to, including not only behavioral and psychological control but also parent-child relationship quality. Second, we examined these parental factors’ effects on the rate of change in adolescent delinquency. Finally, paternal and maternal factors’ relative cross-sectional and longitudinal contributions to adolescent delinquency were also examined. While some previous studies have focused on one or two of these questions among Western adolescents [3,8,32,44], no research has addressed them all together in one single study in Chinese contexts. 

For the level of adolescent delinquency, boys showed a higher initial delinquency level than female peers and this difference remained during the 3-year period, which echoes previous findings on gender differences [31,46]. Besides, a similar increasing trend of delinquency level was observed among boys and girls. Furthermore, children’s gender did not significantly affect the linkages between parenting factors and delinquency, which appeared to be in line with the findings of recent meta-analyses [2,24]. Regarding parental factors’ predictive effect (i.e., the first research question), as expected, behavioral control of both parents and their relationships with children negatively predicted the initial adolescent delinquency level (i.e., Hypotheses 1a, 1b, 1e, and 1f were supported). The findings were congruous with past research which suggested that positive parenting in terms of behavioral control and good parent-child relationship protect children from problem behaviors [2,8,46]. However, the present study did not identify significant predictive effects of both parents’ psychological control on the initial level of adolescent delinquency (i.e., Hypotheses 1c and 1d were not supported). One possible explanation is that the present Chinese adolescents tended not to perceive parental psychological control as negative parenting.

In Western countries where autonomy and individual independence are emphasized, it has been theoretically and empirically verified that paternal psychological control which harms children’s sense of autonomy and self-representation concepts (e.g., self-identity and self-esteem) damages children’s psychological well-being and leads to problem behaviors [2,49]. In contrast, Chinese culture values collectivistic interdependence and family obligations (e.g., respect for parents, filial piety, etc.). Hence, parents’ psychological control might not be as harmful as it is in Western societies. Chinese children who are socialized to comply with traditional values might have more positive feelings toward psychologically controlling parents [65,66]. Indeed, Chinese children are more inclined to perceive parents’ control as a way to show love and concern but less likely to regard authoritarian parenting behaviors as controlling or intrusive [66,67]. Furthermore, past research has suggested that Chinese parents’ dysfunctional parenting did not increase children’s problem behavior [68,69]. Another possibility is that Chinese people may regard certain elements of psychological control (such as guilt induction) as a culturally acceptable way of child rearing [70].

However, contrary to the above thesis, Luebbe et al. [71] found that Chinese parents’ psychological control also result in internalizing problems such as depression and hopelessness in their children. Similarly, Shek and collaborators’ recent work underscored a significant and relatively robust positive relationship between parents’ psychological control and children’s Internet addiction among Chinese adolescents in Hong Kong [23,64]. In addition, the present individual growth curve (IGC) analyses did not reveal a significant predicting effect of psychological control on the initial level of adolescent delinquency. Yet, the regression analyses suggested that there was a positive correlational association between both parents’ psychological control and children’s delinquency, although the effect sizes were not very high. Concerning these findings, we may conjecture that parental psychological control is also likely to negatively affect adolescent development in Chinese communities, but the magnitude of the effect may depend on the indicator of developmental outcome. It is possible that psychological control of Chinese parents has a stronger influence on other developmental outcome measures (e.g., depression and Internet addiction) than on adolescent delinquency. More research is needed to test this possibility by involving a more comprehensive set of measures including both internalizing (e.g., depression) and externalizing problems (e.g., delinquency) and comparing the predictive effects of parental psychological control.

With reference to the second research question, in line with the general consensus, we also observed an increasing trend of adolescent delinquency during junior high school years. Besides, the level of delinquency among boys increased faster than that of girls. This gender difference is also consistent with previous findings [8,72]. However, all the hypotheses relating parental factors to the rate of change in adolescent delinquency were not supported. While maternal behavioral control and relationship between children and both parents did not show significant predictive effects, a higher level of paternal behavioral control was associated with a faster growth in adolescent delinquency, which is discordant with our expectation. This unexpected effect of fathers’ behavioral control was inconsistent with previous findings that parents’ firmer behavioral control or better relationship between father and child predicted a slower increase or a faster decline in adolescent externalizing behavior [3,8]. However, our finding is concordant with some previous research which found that positive parenting characteristics including sufficient behavioral control and better relationship between children and parents predicted a slower drop in adolescent Internet addiction [23,64].

One can argue that high paternal behavioral control’s protective effect may weaken over time, which result in the present association between high baseline paternal behavioral control and faster increasing in adolescent delinquency. It is quite plausible since that the parenting–delinquency link is less significant among older children with the influence of parents weakening as children mature and other social relations exerting greater influences [2,23,73,74]. The less significant effect of paternal behavioral control over time can also be reflected from the present observation that parental factors accounted for less variance in adolescent delinquency at Wave 3 (6.8%) than at Wave 1 (9.0%). Nevertheless, adolescents with stronger baseline paternal behavioral control demonstrated a lower delinquency level across the three waves than other participants with a relatively lower paternal behavioral control, suggesting the critical role of paternal behavioral control. Additionally, the effect of parent-child relationship qualities did not show a similar declining trend as paternal behavioral control. It is possible that the influence of some parental factors (e.g., behavioral control) is more likely to decline than others (e.g., relationship quality) [23]. Given the equivocal findings in the past research and the present study, the predictive effects of parental characteristics on the growth rate of adolescent delinquency are not conclusive. Hence, more replication studies should be carried out in future.

For the third research question that concerned differential cross-sectional and longitudinal predictive effects of different parental factors, three observations can be highlighted. First, for cross-sectional effect, compared with maternal factors, paternal parenting characteristics explained a slightly higher percentage of variance in adolescent delinquency across the three years. However, for longitudinal effects, baseline father-child and mother-child subsystem factors accounted for a similar percentage of variance in future adolescent delinquency. Based on these findings, we contended that paternal impact may be equally strong or even stronger as compared with maternal impact on the development of adolescent delinquency. This interpretation could be compatible with previous findings showing similar or greater effects of paternal parenting [2,32,48,49]. This conclusion makes sense because fathers are less engaged in socializing children but they play a more authoritative and dominant role than mothers in family, especially in Chinese communities [75]. 

The second observation is that paternal impact may be stronger in one specific aspect, while there will be a greater maternal impact in other aspects. Specifically, the present study found that at Waves 1 and 2, fathers’ behavioral control was more influential than mothers’ behavioral control whereas mothers’ relationship with children exerted stronger impact than father-child relationship. Such differences became insignificant at Wave 3, possibly due to gradually decreased parental influence on children as aforementioned. The findings indicate that relative maternal and paternal impacts should be considered in relation to a specific parental factor. For example, Bean et al. [76] reported a significant predictive effect of fathers’, but not mothers’ support on children’s depression and a significant effect of mothers’, but not fathers psychological control on children’s academic achievement. Recently, Shek et al. [64] found that fathers were more influential on adolescent Internet addiction by behavioral control while mothers were more influential by psychological control. This pattern of different maternal impact versus paternal impact can help explain the mixed findings in past studies as most of them only investigated one aspect of parenting such as psychological control [77]. 

Finally, parent-child relationship qualities, particularly mother-child relationship quality showed the most robust longitudinal predictive effect on adolescent delinquency. This observation echoes one finding of present IGC modeling that the baseline parent-child relationship qualities did not predict the growth rate of delinquency (i.e., the effect of baseline parent-child relationship qualities on delinquency level did not decline over time). These findings confirm the assertion that not only parents’ monitoring and control of adolescent activities, but also children’s satisfaction with control and willingness to communicate, prevent deviant behaviors. It supports scholars’ previous contention that a good relationship between parents and children operates preventively and is “a two-way process including both the parents’ solicitation or knowledge and control of their children’s behavior and the children’s willingness to make their parents part of their lives” [39] (pp. 1083). Even more, without a good parent-child relationship, children may perceive strong parental behavioral control as intrusive and gain less positive influence or even negative influence from parental behavioral control [35,41]. Thus, our findings help draw further attention to the need for including parent-child relationships when investigating parental control. 

The findings of this study have implications for the development of prevention or intervention parent training programs. Notably, there is a dearth of parent training programs focusing on delinquency in Hong Kong. In Piquero et al.’s [78] study, 78 studies that evaluated the parent training programs on delinquency were reviewed, but only one study was conducted in Hong Kong. Because interventions need to be guided by theory and based on valid empirical research findings, our findings on the associations between multiple parenting characteristics and adolescent delinquency serve as the theoretical framework for the parent training in Hong Kong. First, the significant effects of parental behavioral control, particularly paternal behavioral control, suggest that parent training programs should train parents to rule and actively monitor their children so as to enhance parents’ knowledge on children’s whereabouts and conduct. Second, given that parent-child relationship demonstrated robust effects on delinquency, parent training should also promote parental skills in improving the relationship, communication, and mutual trust with children, which in turn enhance children’s self-disclosure. Finally, training programs should involve not only mothers but also fathers. This is consistent with previous findings that parent training brought more benefits to children if fathers also attended the training [79].

Despite the pioneering nature of the study and its theoretical and practical implications, it has several limitations. First, the present study relied on adolescent self-report. Past research suggested that effect sizes of the links between parenting and adolescent problems depend on the informants employed [2,80]. It is commented that children tend to overestimate the negative features of their parents while parents are more inclined to overestimate positive qualities of their own parenting practice [2]. While it remains unknown as to which informants evaluate parenting most objectively, child report measures are widely used, especially in longitudinal research. It is arguable that children know themselves better than others and what actually matters are children’s perception and experience. Another related issue is social desirability associated with self-report measures. While it is difficult to eliminate social desirability bias, the present study strived to reduce it as much as possible through emphasizing anonymity and instructing the participants to give honest answers. Nevertheless, future research will certainly benefit from collecting data through multiple informants (e.g., children, parents, teachers and peers) as well as different measuring methodologies (e.g., self-report and objective observation). Third, internalizing problems such as depression and externalizing problems such as delinquency can co-occur in a homotypic (*either* comorbid internalizing *or* comorbid externalizing problems within individuals) or heterotypic (comorbidity of *both* internalizing *and* externalizing problems within individuals) way [12,81]. However, the present study only measured delinquency. Hence, we are not sure about the relationships between adolescent delinquency and other emotional or behavioral problems; and whether such relationships would moderate the parenting effect. Future study should involve more forms of adolescent problems and test their interactions. Finally, our study only involved Hong Kong Chinese adolescents. To improve the universality of the present findings, future research should address relevant research questions in other Chinese subcultures such as mainland China and Taiwan, and compare findings obtained from different samples. 

## 5. Conclusions

Despite the above limitations, our findings add great value to the existing literature and indicate that it is essential to differentiate between: (1) adolescent delinquency level and its change rate over time; (2) different aspects of parent-child dyadic factors; and (3) paternal and maternal factors. The present findings also have important implications for designing parent training programs on adolescent delinquency. It is suggested to involve both parties of parents and focus on not only active parental monitoring, but also the improvement of relationship and trust between parents and children.

## Figures and Tables

**Table 1:** Hypotheses of the present study.

Hypotheses	Brief Descriptions	Supported by Results (Yes/No)
1a	Paternal behavioral control negatively predicts the initial level of adolescent delinquency	Yes
1b	Maternal behavioral control negatively predicts the initial level of adolescent delinquency	Yes
1c	Paternal psychological control positively predicts the initial level of adolescent delinquency	No
1d	Maternal psychological control positively predicts the initial level of adolescent delinquency	No
1e	Father-child relationship quality negatively predicts the initial level of adolescent delinquency	Yes
1f	Mother-child relationship quality negatively predicts the initial level of adolescent delinquency	Yes
2a	Higher paternal behavioral control will predict a slower increase in adolescent delinquency	No
2b	Higher maternal behavioral control will predict a slower increase in adolescent delinquency	No
2c	Higher paternal psychological control will predict a faster increase in adolescent delinquency	No
2d	Higher maternal psychological control will predict a faster increase in adolescent delinquency	No
2e	A better father-child relationship quality will predict a slower increase in adolescent delinquency	No
2f	A better mother-child relationship quality will predict a slower increase in adolescent delinquency	No
3a	Paternal factors are more influential than maternal factors in shaping adolescent delinquency	Yes
3b	Maternal factors are more influential than paternal factors in influencing adolescent delinquency	No

**Table 2:** Reliability of scales across the three waves.

Scale	Number of Items	Wave	Cronbach’s *α*	Mean Inter-Item Correlation
Delinquency	12	Wave 1	0.81	0.27
		Wave 2	0.84	0.33
		Wave 3	0.79	0.25
Father–Child Subsystem Quality Scale	17			
Paternal behavioral control	7	Wave 1	0.89	0.54
		Wave 2	0.89	0.54
		Wave 3	0.89	0.53
Paternal psychological control	4	Wave 1	0.80	0.50
		Wave 2	0.83	0.54
		Wave 3	0.86	0.61
Father-child relational quality	6	Wave 1	0.90	0.60
		Wave 2	0.91	0.62
		Wave 3	0.90	0.62
Mother–Child Subsystem Quality Scale	17			
Maternal behavioral control	7	Wave 1	0.90	0.55
		Wave 2	0.89	0.54
		Wave 3	0.89	0.54
Maternal psychological control	4	Wave 1	0.85	0.59
		Wave 2	0.88	0.64
		Wave 3	0.89	0.67
Mother-child relational quality	6	Wave 1	0.91	0.63
		Wave 2	0.91	0.64
		Wave 3	0.90	0.62

**Table 3:** Correlations among variables.

Variables		Range	*M*	*SD*	1	2	3	4	5	6	7	8	9
1.	Gender ^a^				--								
2.	PBC	1–4	2.56	0.66	0.03	--							
3.	PPC	1–4	2.24	0.70	0.13 ***	0.18 ***	--						
4.	FCRQ	1–4	2.80	0.68	0.003	0.67 ***	−0.08 ***	--					
5.	MBC	1–4	3.03	0.62	−0.06 **	0.43 ***	0.08 ***	0.35 ***	--				
6.	MPC	1–4	2.31	0.77	0.07 ***	−0.003	0.49 ***	−0.10 ***	0.09 ***	--			
7.	MCRQ	1–4	3.05	0.66	−0.05 ***	0.37 ***	−0.001	0.44 ***	0.68 ***	−0.16 ***	--		
8.	DE1	0–6	0.36	0.39	0.06 **	−0.23 ***	0.04	−0.23 ***	−0.18 ***	0.07 ***	−0.24 ***	--	
9.	DE2	0–6	0.43	0.47	0.07 ***	−0.29 ***	0.04 *	−0.20 ***	−0.17 ***	0.06 **	−0.21 ***	0.53 ***	--
10.	DE3	0–6	0.44	0.48	0.11 ***	−0.14 ***	0.03	−0.17 ***	−0.14 ***	0.04 *	−0.17 ***	0.44 ***	0.57 ***

Note. The correlational patterns between parental factors measured at different waves and other variables were the same, so only the results on Wave 1 parental factors were presented in the table due to space limit. M = Mean; SD = Standard deviation; PBC = paternal behavioral control; PPC = paternal psychological control; FCRQ = father-child relational quality; MBC = maternal behavioral control; MPC = maternal psychological control; MCRQ = mother-child relational quality; DE1 = delinquency at Wave 1; DE2 = delinquency at Wave 2; DE3 = delinquency at Wave 3. ^a^ Female = −1, Male = 1; * *p* < 0.05; ** *p* < 0.01; *** *p* < 0.001.

**Table 4:** Results of individual growth curve (IGC) models with Level-1 predictors for adolescent delinquency (Waves 1–3).

		Model 1		Model 2		Model 2 (Male)		Model 2 (Female)	
		Estimate	SE	Estimate	SE	Estimate	SE	Estimate	SE
Fixed effects									
Intercept	*β*_0*j*_								
Intercept	*γ*_00_	0.305 ***	0.0044	0.281 ***	0.0048	0.296 ***	0.0070	0.267 ***	0.0064
Linear Slope	*β*_1*j*_								
Time	*γ*_10_			0.024 ***	0.0026	0.030 ***	0.0040	0.017 ***	0.0034
Random effects									
Level 1 (within)									
Residual	*r_ij_*	0.0342 ***	0.0007	0.0306 ***	0.0008	0.0349 ***	0.0014	0.0265 ***	0.0010
Level 2 (between)									
Intercept	*u*_0*j*_	0.0393 ***	0.0014	0.0351 ***	0.0018	0.0360 ***	0.0028	0.0336 ***	0.0023
Time	*u*_1*j*_			0.0030 ***	0.0007	0.0038 ***	0.0011	0.0020 *	0.0007
Fit statistics									
Deviance		−328.69		−489.09		205.12		−792.55	
AIC		−322.69		−477.09		217.12		−780.55	
BIC		−301.72		−435.16		254.84		−742.74	
df		3		6		6		6	

Note. Model 1 = unconditional mean model; Model 2 = unconditional linear growth model. SE = Standard error; AIC = Akaike information criterion; BIC = Bayesian information criterion; df = degree of freedom. * *p* < 0.05; *** *p* < 0.001.

**Table 5:** Results of IGC models with Level-2 predictors for adolescent delinquency (Waves 1–3).

		Model 3		Model 4a	
		Estimate	SE	Estimate	SE
Fixed effects					
Intercept	*β*_0*j*_				
Intercept	*γ*_00_	0.282 ***	0.0048	0.282 ***	0.0046
Gender ^a^	*γ*_01_	0.014 **	0.0048	0.015 **	0.0046
Paternal behavioral control	*γ*_02_			−0.052 ***	0.0051
Maternal behavioral control	*γ*_03_			−0.026 ***	0.0051
Linear Slope	*β*_1*j*_				
Intercept	*γ*_10_	0.024 **	0.0026	0.0237 ***	0.0026
Gender ^a^	*γ*_11_	0.007 **	0.0026	0.0070 **	0.0026
Paternal behavioral control	*γ*_12_			0.0085 **	0.0029
Maternal behavioral control	*γ*_13_			0.0006	0.0029
Random effects					
Level 1 (within)					
Residual	*r_ij_*	0.0306 ***	0.0008	0.0306 ***	0.0008
Level 2 (between)					
Intercept	*u*_0*j*_	0.0348 ***	0.0018	0.0304 ***	0.0017
Time	*u*_1*j*_	0.0029 ***	0.0007	0.0028 ***	0.0007
Fit statistics					
Deviance		−519.022		−735.552	
AIC		−503.022		−711.552	
BIC		−447.118		−627.695	
df		8		12	

Note. Model 3 = conditional growth curve model (only with gender); Model 4 = conditional growth curve model (adding parental behavioral control). ^a^ Female = −1, Male = 1. SE = Standard error; AIC = Akaike information criterion; BIC = Bayesian information criterion; df = degree of freedom. ** *p* < 0.01. *** *p* < 0.001.

**Table 6:** Results of IGC models with Level-2 predictors for adolescent delinquency (Waves 1–3).

		Model 3		Model 4b	
		Estimate	SE	Estimate	SE
Fixed effects					
Intercept	*β*_0*j*_				
Intercept	*γ*_00_	0.282 ***	0.0048	0.282 ***	0.0047
Gender ^a^	*γ*_01_	0.014 **	0.0048	0.013 **	0.0048
Paternal psychological control	*γ*_02_			0.001	0.0054
Maternal psychological control	*γ*_03_			0.017 **	0.0054
Linear Slope	*β*_1*j*_				
Intercept	*γ*_10_	0.024 **	0.0026	0.024 ***	0.0026
Gender ^a^	*γ*_11_	0.007 **	0.0026	0.007	0.0026
Paternal psychological control	*γ*_12_			0.0008	0.00297
Maternal psychological control	*γ*_13_			−0.0037	0.00296
Random effects					
Level 1 (within)					
Residual	*r_ij_*	0.0306 ***	0.0008	0.0306 ***	0.0008
Level 2 (between)					
Intercept	*u*_0*j*_	0.0348 ***	0.0018	0.0345 ***	0.0018
Time	*u*_1*j*_	0.0029 ***	0.0007	0.0029 ***	0.0007
Fit statistics					
Deviance		−519.022		−532.195	
AIC		−503.022		−508.195	
BIC		−447.118		−424.338	
df		8		12	

Note. Model 3 = conditional growth curve model (only with gender); Model 4 = conditional growth curve model (adding parental psychological control). ^a^ Female = −1, Male = 1. SE = Standard error; AIC = Akaike information criterion; BIC = Bayesian information criterion; df = Degree of freedom. ** *p* < 0.01. *** *p* < 0.001.

**Table 7:** Results of IGC models with Level-2 predictors for adolescent delinquency (Waves 1–3).

		Model 3		Model 4c	
		Estimate	SE	Estimate	SE
Fixed effects					
Intercept	*β*_0*j*_				
Intercept	*γ*_00_	0.282 ***	0.0048	0.282 ***	0.0045
Gender ^a^	*γ*_01_	0.014 **	0.0048	0.013 **	0.0046
Father-child relational quality	*γ*_02_			−0.039 ***	0.0051
Mother-child relational quality	*γ*_03_			−0.046 ***	0.0051
Linear Slope	*β*_1*j*_				
Intercept	*γ*_10_	0.024 **	0.0026	0.024 ***	0.0026
Gender ^a^	*γ*_11_	0.007 **	0.0026	0.007 **	0.0026
Father-child relationship	*γ*_12_			0.0018	0.0029
Mother-child relationship	*γ*_13_			0.0054	0.0029
Random effects					
Level 1 (within)					
Residual	*r_ij_*	0.0306 ***	0.0008	0.0306 ***	0.0008
Level 2 (between)					
Intercept	*u*_0*j*_	0.0348 ***	0.0018	0.0296 ***	0.0017
Time	*u*_1*j*_	0.0029 ***	0.0007	0.0029 ***	0.0007
Fit statistics					
Deviance		−519.022		−782.566	
AIC		−503.022		−758.566	
BIC		−447.118		−674.709	
df		8		12	

Note. Model 3 = conditional growth curve model (only with gender); Model 4 = conditional growth curve model (adding parent-child relational qualities). ^a^ Female = −1, Male = 1. SE = Standard error; AIC = Akaike information criterion; BIC = Bayesian information criterion; df = degree of freedom. ** *p* < 0.01. *** *p* < 0.001.

**Table 8:** Concurrent predicting effects of parent-child subsystem qualities on delinquency.

Model	Predictors	Wave 1 Delinquency ^a^			Wave 2 Delinquency ^b^			Wave 3 Delinquency ^c^		
		*β*	*t*	Cohen’s *f*^2^	*β*	*t*	Cohen’s *f*^2^	*β*	*t*	Cohen’s *f*^2^
1	Gender ^d^	0.06	2.83 ***	0.004	0.07	3.51 **	0.005	0.12	5.68 ***	0.014
	*R*^2^ change	0.004			0.005			0.014		
	*F* change	8.03 **			12.29 ***			32.22 ***		
2	PBC	−0.17	−5.29 ***	0.014	−0.16	−5.83 ***	0.014	−0.17	−5.83 ***	0.015
	PPC	0.05	2.46 *	0.003	0.06	3.07 **	0.004	0.08	3.51 ***	0.005
	FCRQ	−0.12	−4.13 ***	0.008	−0.14	−5.04 ***	0.011	−0.07	−2.53 *	0.003
	*R*^2^ change	0.071			0.079			0.054		
	*F* change	55.95 ***			66.26 ***			44.76 ***		
3	MBC	−0.05	−1.66	0.001	−0.07	−2.67 **	0.003	−0.13	−4.79 ***	0.009
	MPC	0.03	0.03	0.001	0.06	2.68 **	0.003	0.09	4.22 ***	0.007
	MCRQ	−0.21	−0.21 ***	0.023	−0.20	−7.25 ***	0.021	−0.09	−3.27 **	0.004
	*R*^2^ change	0.061			0.068			0.049		
	*F* change	51.54 ***			60.58 ***			42.30 ***		
4	PBC	−0.15	−4.92 ***	0.012	−0.15	−4.96 ***	0.011	−0.13	−4.26 ***	0.008
	PPC	0.06	2.23 *	0.003	0.06	2.41 *	0.003	0.05	1.86	0.002
	FCRQ	−0.06	−1.94	0.002	−0.07	−2.29 *	0.002	−0.04	−1.39	0.001
	MBC	0.01	0.31	0.000	0.01	0.16	0.000	−0.06	−2.01 *	0.002
	MPC	0.00	0.02	0.000	0.02	0.64	0.000	0.06	2.47 *	0.003
	MCRQ	−0.16	−5.17 ***	0.013	−0.17	−5.82 ***	0.015	−0.07	−2.25 ***	0.002
	*R*^2^ change	0.090			0.100			0.068		
	*F* change	34.39 ***			42.99 ***			27.82 ***		

*Note.* For Model 2–4, gender was controlled. ^a^ Parental factors measured at Wave 1 were used; ^b^ Parental factors measured at Wave 2 were used; ^c^ Parental factors measured at Wave 3 were used; ^d^ Female = −1, Male = 1. PBC = paternal behavioral control; PPC = paternal psychological control; FCRQ = father-child relational quality; MBC = maternal behavioral control; MPC = maternal psychological control; MCRQ = mother-child relational quality. * *p* < 0.05. ** *p* < 0.01. *** *p* < 0.001.

**Table 9:** Longitudinal predicting effects of the parent-child subsystem qualities on delinquency.

Model	Predictors	Wave 2 Delinquency			Wave 3 Delinquency		
		*β*	*t*	Cohen’s *f*^2^	*β*	*t*	Cohen’s *f*^2^
1	Gender ^a^	0.07	3.51 ***	0.005	0.12	5.68 ***	0.014
	*R*^2^ change	0.005			0.014		
	*F* change	12.29 ***			32.22 ***		
2	PBC	−0.13	−4.30 ***	0.008	−0.06	−1.83	0.002
	PPC	0.05	2.21 *	0.002	0.01	0.42	0.000
	FCRQ	−0.11	−3.31 ***	0.006	−0.14	−4.65 ***	0.010
	*R*^2^ change	0.048			0.034		
	*F* change	37.39 ***			25.23 ***		
3	MBC	−0.06	−2.30 *	0.002	−0.05	−1.70	0.001
	MPC	0.04	1.90	0.001	0.02	0.97	0.000
	MCRQ	−0.16	−5.62 ***	0.013	−0.14	−4.76 ***	0.010
	*R*^2^ change	0.046			0.030		
	*F* change	36.06 ***			25.10 ***		
4	PBC	−0.11	−3.57 ***	0.006	−0.03	−0.92	0.000
	PPC	0.05	2.07 *	0.002	0.01	0.51	0.000
	FCRQ	−0.06	−1.82 ^^^	0.002	−0.10	3.12 **	0.005
	MBC	−0.02	−0.62	0.000	−0.03	−0.95	0.000
	MPC	−0.002	−0.09	0.000	0.00	0.06	0.000
	MCRQ	−0.12	−3.76 ***	0.007	−0.11	−3.63 **	0.006
	*R*^2^ change	0.061			0.046		
	*F* change	23.17 ***			16.76 ***		

Note. For Model 2–4, gender was controlled; parental factors measured at Wave 1 were used as predictors; ^a^ Female = −1, Male = 1. PBC = paternal behavioral control; PPC = paternal psychological control; FCRQ = father-child relational quality; MBC = maternal behavioral control; MPC = maternal psychological control; MCRQ = mother-child relational quality. ^ *p* < 0.10. * *p* < 0.05. ** *p* < 0.01. *** *p* < 0.001.
